# Proteome TopFIND 3.0 with TopFINDer and PathFINDer: database and analysis tools for the association of protein termini to pre- and post-translational events

**DOI:** 10.1093/nar/gku1012

**Published:** 2014-10-20

**Authors:** Nikolaus Fortelny, Sharon Yang, Paul Pavlidis, Philipp F. Lange, Christopher M. Overall

**Affiliations:** 1 Department of Biochemistry and Molecular Biology, University of British Columbia, Vancouver, British Columbia, Canada; 2 Department of Oral Biological and Medical Sciences, University of British Columbia, Vancouver, British Columbia, Canada; 3 Centre for Blood Research, University of British Columbia, Vancouver, British Columbia, Canada; 4 Centre for High Throughput Biology, University of British Columbia, Vancouver, British Columbia, Canada; 5 Department of Computer Science, University of British Columbia, Vancouver, British Columbia, Canada; 6 Department of Statistics, University of British Columbia, Vancouver, British Columbia, Canada; 7 Department of Psychiatry, University of British Columbia, Vancouver, British Columbia, Canada

## Abstract

The knowledgebase TopFIND is an analysis platform focussed on protein termini, their origin, modification and hence their role on protein structure and function. Here, we present a major update to TopFIND, version 3, which includes a 70% increase in the underlying data to now cover a 90 696 proteins, 165 044 N-termini, 130 182 C-termini, 14 382 cleavage sites and 33 209 substrate cleavages in *H. sapiens*, *M. musculus, A. thaliana, S. cerevisiae* and *E. coli*. New features include the mapping of protein termini and cleavage entries across protein isoforms and significantly, the mapping of protein termini originating from alternative transcription and alternative translation start sites. Furthermore, two analysis tools for complex data analysis based on the TopFIND resource are now available online: TopFINDer, the TopFIND ExploRer, characterizes and annotates proteomics-derived N- or C-termini sets for their origin, sequence context and implications for protein structure and function. Neo-termini are also linked to associated proteases. PathFINDer identifies indirect connections between a protease and list of substrates or termini thus supporting the evaluation of complex proteolytic processes *in vivo*. To demonstrate the utility of the tools, a recent N-terminomics data set of inflamed murine skin has been re-analyzed. In re-capitulating the major findings originally performed manually, this validates the utility of these new resources. The point of entry for the resource is http://clipserve.clip.ubc.ca/topfind from where the graphical interface, all application programming interfaces (API) and the analysis tools are freely accessible.

## INTRODUCTION

Genetic information typically results in many protein species differing in amino acid sequence or by modification of individual amino acids. Pre- and post-translational processes can result in protein species that differ from other species encoded by the same gene in the extent of their sequence. Such protein species can have fundamentally different properties due to differences in primary structure including different domain structure, linear motifs, post-translational modification sites, as well as protein and ligand binding sites. This can radically change the function of the protein, its location within a cell, whether it is exported or not to function extracellularly. The start and end of a protein chain, the N- and C-termini, respectively, are a particularly defining feature marking the extent of the primary structure and thus the functional competence of the protein. Since the chemistries of the protein termini differ from the amino acid side chains, protein termini can also undergo specific post-translational modifications that in turn can direct function. Thus, knowledge of protein termini is an essential facet in understanding the structure and function relationships of proteins and hence networks of proteins.

**Figure 1.**
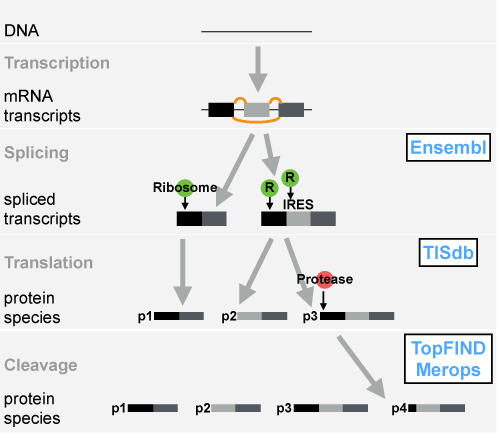
Biological processes leading to differences in termini in proteins and databases containing corresponding information.

Three independent processes form protein species that differ in the extent of their sequence and hence termini. First, RNA splicing can lead to the selection of alternative exons encoding the N- or C-terminus (Figure 1). Second, use of alternative translation initiation sites leads to protein species with either shorter or longer sequence and hence alternate protein N-termini (Figure 1). Third, post-translational modification by proteolytic processing truncates the protein and leads to the formation of shorter stable protein chains with a unique neo N- or neo C-terminus (Figure 1) (1,2). Recent analyses show that proteolytic processing of proteins *in vivo* in tissues (3) or specialized enucleate cells, the erythrocyte (4) and platelet (5), is remarkably high at 49%, 68% and 77%, respectively. Such analyses reveal the formation of stable protein species by proteolytic processing as a pervasive phenomenon in proteomes and hence one that needs to be considered in interpreting biological processes.

Although proteolytic processing by proteases, also known as peptidases, in principle affects every protein in the proteome, it is a generally underappreciated post-translational modification (2). Endopeptidases cleave a protein at a precise position in the sequence whereas exopeptidases remove one and two amino acids from the end of the protein chain. If trimming continues in a processive manner this is termed ‘ragging’. Through these three main specific protein modifications, the start and end position of a protein has been found at any point in the genome encoded protein sequence even to within 20% of the protein length from the C-terminus (6). The known functional implications of proteolysis include conventional protein maturation, e.g. removal of N-terminal methionine, signal and transit peptides, as well as specific chain cleavages during protein maturation, or cleavages after disulphide bridges form protein homodimers or heteromers. Precise proteolytic processing also generates bioactive peptides from parent chains, e.g. bradykinin and many hormones, and can act at different stages in the life of a protein to modulate protein function, localization and membrane protein shedding.

The chemical nature and length of different protein N- and C-termini not only conveys different functional properties, such as altered receptor binding to switch from agonist to antagonist as exemplified by the truncation of four amino acids only from the N-terminus of CC chemokines e.g. CCL2 and CCL7 (1,7). Most prominent chemical changes include N-terminal acetylation which regulates protein stability and half-life, either destabilizing the protein (8) or increasing stability in a sequence dependent manner (4). Other notable examples include N-myristoylation and N-palmitoylation which regulate protein trafficking and membrane localization (9,10). Modifications of the C-terminus are less well understood due to the difficulty in identifying C-termini by sequencing or in their enrichment, but are equally relevant as for example the carboxyl methylation of phosphoprotein phosphatase 2A which is a prerequisite for the association with its Bα subunit (11).

Whereas the impact of proteases on protein termini and function is of great biological relevance *in vivo* studies of proteases and their substrates are complicated by the deeply connected and dynamic interactions of proteases in cells and tissues (12). In particular, proteases interact to form an interconnected network termed the protease web (12) by cleaving other proteases or protease inhibitors. Thereby a protease indirectly influences the cleavage of substrates of downstream proteases in addition to the direct substrate repertoire. Such interactions complicate the differentiation between direct and indirect cleavage events and thus hamper the assignment of proteases to substrates in *in vivo* studies. In our recent comprehensive investigation of the properties of the protease web we showed that computational modeling of existing cleavage and inhibition information can greatly assist in distinguishing direct and indirect effects and can be used to assist in assigning proteases to cleavage events observed *in vivo* (12). However, tools for performing this routinely are desperately needed in order to interpret biological phenomena, knockout mouse models and for drug target validation.

In view of the added complexity arising from altered termini position and nature we developed TopFIND (6,13) to comprehensively integrate data on protein termini and their formation by proteolytic processing as well as to associate shortened protein chains with relevant information on protein function. A web interface and application programming interfaces (API) enable manual and automated data retrieval and analysis from TopFIND. The web interface is protein-centric with a dedicated summary page for each protein. Protein-pages contain general protein specific information and a variety of information related to the termini of the protein, position-specific information of domains and features, sites of proteolytic processing and positions of protein termini and mutation or single nucleotide polymorphism (SNP) sites altering the protein sequence at or in the neighborhood of cleavage sites (6). Proteases and protease inhibitors are further annotated by dynamically calculated cleavage site specificities and their connectivity in the protease web. For each observed terminus or cleavage the underlying evidence including information on confidence, biological relevance, experimental conditions and publications is displayed. A powerful filter enables efficient selection of specific evidence parameters, such as a confidence cut-off or source laboratory, enabling users to focus on a subset of termini and cleavages.

Here we present the next major release of TopFIND version 3.0 in which we addressed three major limitations and user needs. First, we now account for all biological processes leading to the formation of alternate termini in all isoforms including alternative translation and splicing. Second, by creating the analysis software TopFIND ExploRer (TopFINDer) we have enabled researchers to annotate and statistically evaluate large-scale proteomics experiments in view of the TopFIND resource. Third, with PathFINDer we developed the first publicly available tool to identify putative indirect proteolytic effects from *in vivo* proteomics data by placing proteins in the context of the proteolytic network (the extension of the protease web generated by adding known and MEROPS annotated protease substrates) (12) and identifying indirect connections from a query protease to the protein using graph path finding. With these new tools, TopFIND 3.0 now addresses and greatly facilitates solving the hardest problem in current protease research, the identification of the cognate protease responsible for a given cleavage event from a complex *in vivo* sample.

## MATERIALS AND METHODS

TopFIND is developed in Ruby with a MySQL database backend and a web application frontend developed on a Rails framework as described previously (6). To annotate termini inferred from alternative transcripts, human and mouse Ensembl (14) protein (ENSP) sequences were downloaded in FASTA format from http://uswest.Ensembl.org/info/data/ftp/index.html. The 20 first and last amino acids of each protein sequence were mapped to the corresponding UniProt (15) sequence to annotate the position of the new N- and C-termini, respectively. To annotate N-termini derived from alternative translation start sites, human and mouse TISdb (16) files were downloaded from http://tisdb.human.cornell.edu/download/. The nucleic acid sequences were retrieved using the RefSeq ID and BioMart, they were then translated *in silico*, and finally 20 amino acids from the indicated start position of the translated sequence were mapped to the protein sequence from UniProt to annotated N-terminus. We developed TopFINDer to show protease enrichment *p*-values that were obtained using a Fisher exact test. The background for this test is made up of cleavages on the proteins from the list by the proteases identified by TopFINDer. TopFINDer then calculates a *q*-value using Benjamini-Hochberg multiple testing correction. Icelogos are created using http://iomics.ugent.be/icelogoserver. Graphical representations of the connections identified by PathFINDer are plotted using graphviz (http://www.graphviz.org/). Mapping of orthologous proteins between *homo sapiens* and *mus musculus* for PathFINDer was derived from the InParanoid database (17) version 8.0. The example data set was taken from data sheet 8 from the supplementary tables published by auf dem Keller *et al.* (3). All peptides were run in TopFINDer with default settings. Peptides with a log_2_ higher or lower than 1.19 (cutoff defined in the original paper) were run in PathFINDer with MMP2 (P33434) as a query protease and using the human network.

## CHANGES TO THE DATABASE CONTENT

### Improved isoform handling

In previous versions of TopFIND we limited the annotation of termini to the canonical isoform as defined by UniProt (15). We have now extended the functionality in two ways. First, in addition to the main entry for the canonical isoform we now provide a full entry with accompanying web page for each individual isoform. Second, in addition to the original isoform-specific annotation we now map every entry to the corresponding isoforms using exact alignment of the local sequence context of the 20 amino acids following (N-terminus), preceding (C-terminus) or surrounding (cleavage) the reported position. Entries derived from isoform mapping are clearly marked and associated with respective evidence stating and linking to the original observation of the inferred terminus.

### Additional protein termini originating from alternative splicing and alternative translation

We incorporated termini entries and corresponding evidence into TopFIND for termini resulting from experimentally observed alternative mRNA transcripts as annotated by Ensembl (14). We collected protein-coding transcripts and mapped their N- and C-termini to protein sequences. Thereby evidence for 11 809 and 6676 termini were added for human and mouse, respectively (Table 1).

**Table 1.** Counts of non-canonical termini evidenced by alternative splicing evidence (from Ensembl) or by alternative translation (from TISdb)

		Non-canonical termini		
Terminus type	Biological process	Human	Mouse	SUM
N-termini	Alternative splicing	3141	1390	4531
	Alternative translation	439	1437	1876
C-termini	Alternative splicing	8229	3849	12 078
	Alternative translation	0	0	0
Total		11 809	6676	18 485

We also added N-termini resulting from alternative translation initiation. Alternative translation of proteins is the result of Internal Ribosome Entry Sites (IRES) or leaky ribosome scanning (16) and is commonly probed by Global Translation Initiation Sequencing (GTI-Seq) where a translation elongation inhibitor is added to a sample and the RNA found with the bound ribosome is sequenced. We integrated this information from TISdb (16), which represents the gold standard database for alternative translation initiation. To date TopFIND incorporates 439 human and 1,437 mouse N-termini based on evidence from alternative translation reported in TISdb. However, we expect these numbers to dramatically increase in the next few years with the increase in proteogenomics studies.

## NEW INTERFACES AND BACK END FOR PROTEOMICS DATA ANALYSIS

### TopFINDer—the TopFIND ExploreR

**Figure 2.**
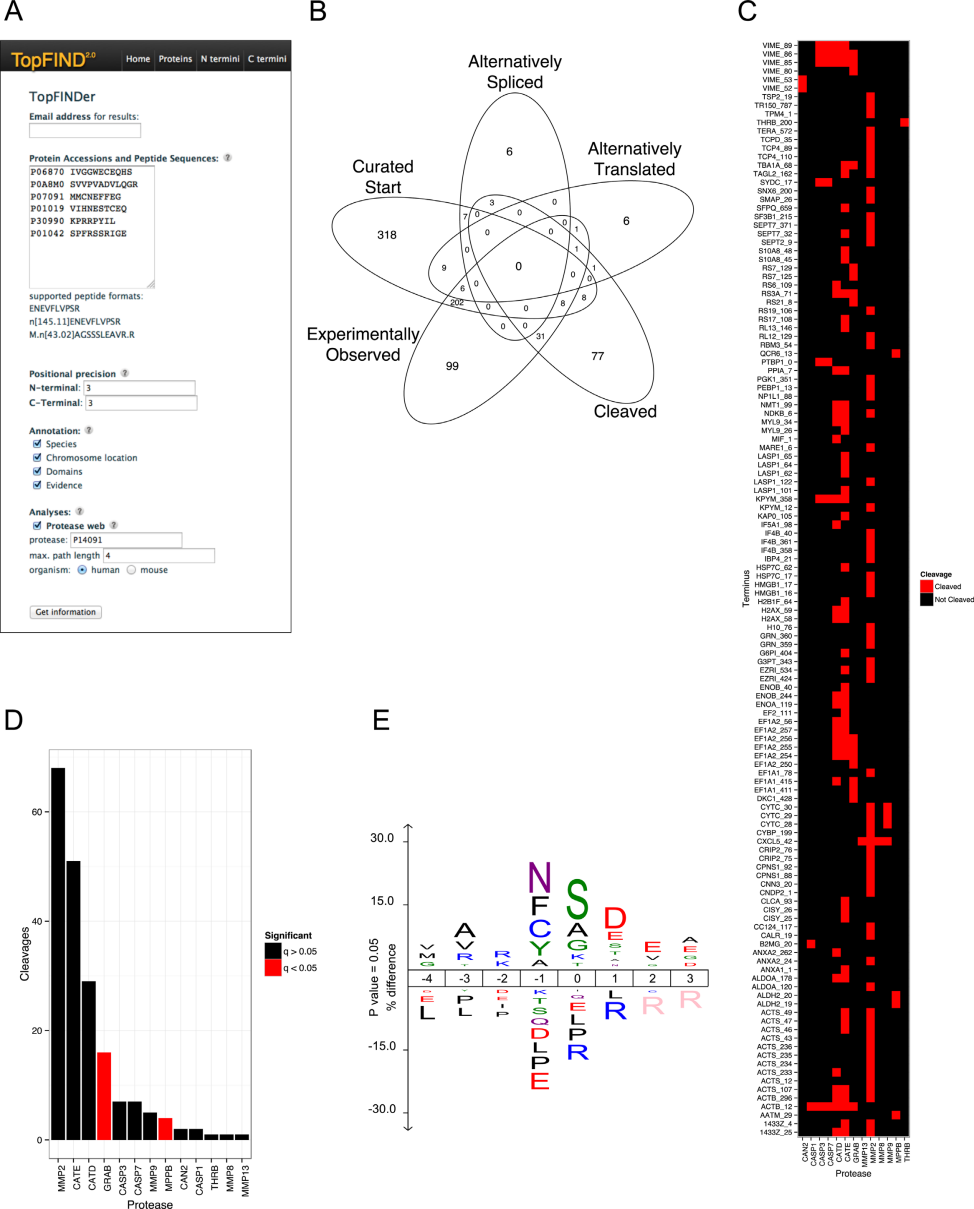
Input and output of TopFINDer. (**A**) Input mask within the TopFIND web interface. (**B**) Venn diagram showing the overlap of termini evidences retrieved from TopFIND for a list of proteins. Evidence is either UniProt annotated terminus (Curated Start), terminus of an isoform derived from alternative splicing (Alternatively Spliced), or from alternative translation (Alternatively Translated), from cleavage (Cleaved), or a terminus observed in a non-protease related terminomics experiment (Experimentally Observed). (**C**) Matrix of substrates and proteases indicating cleavage of substrates. Fields are red where there is cleavage and black where there is none known between the protease and the substrate at this position. The *y*-axis shows the protein identifier and the position of each terminus. (**D**) Barplot showing the number of cleavages of each protease in the list. Bars of proteases whose cleavages are enriched in the list are in red, others in blue. (**E**) IceLogo of the sequences in the list.

A frequently requested functionality is to facilitate information retrieval for lists of thousands of proteins commonly generated by proteomics experiments. Here, we extended TopFIND with such a powerful analysis tool we call TopFINDer. TopFINDer enables analysis and functional annotation of protein N- or C-termini sets based on TopFIND data. From a list of protein identifiers and their terminal amino acid sequences as input (Figure 2A) TopFINDer returns comprehensive protein and terminus related (position-specific) information and analyses based on TopFIND data. The results are sent by email and contain a table (in a EXCEL compatible tab delimited text file) with general annotation of each protein (e.g. Supplementary Table S1) and position-specific information: for each identified terminus, TopFINDer reports the position of the terminus relative to the genome encoded sequence as well as the sequence context surrounding the terminus and evidences for the terminus from TopFIND. Important new information is provided including evidences for their classification by their origin as (i) termini inferred from alternative splicing derived protein isoforms in Ensembl or UniProt, (ii) N-termini inferred from alternative translation, (iii) termini inferred from cleavage together with the associated proteases, (iv) status as UniProt annotated canonical protein termini and (v) termini observed experimentally but without a known protease responsible for the cleavage.

Thoughtful analyses can then be performed by comparing each observed terminus in the list to evidences for this terminus in TopFIND. Thus, the biological relevance of the terminus can be assessed immediately and the biological process generating the protein terminus can be inferred. TopFINDer also displays domains and features N-terminal and C-terminal to the identified terminus as well as features located at the position of the terminus, allowing for inference of the impact on protein function. TopFINDer also allows for the retrieval of information in the proximity of the query terminus using a user definable N- and C-terminal extension of up to 10 amino acids from the terminus position. Thereby, processes such as ragging that lead to nearby but different termini can be accounted for by the original process and included in the analyses.

In addition to annotation, TopFINDer calculates summary and enrichment statistics for each of the submitted termini that TopFINDer classifies as described above. The overlap and relative distribution of these groups is visualized in a Venn diagram (Figure 2B). Of great use to the protease community, termini originating from proteolytic processing are assigned to proteases already annotated by TopFIND to mediate the specific cleavage. These data are visualized in a clustered matrix with substrates on the *y*-axis and proteases on the *x*-axis (Figure 2C) as well as a histogram of counts of cleavages per protease (Figure 2D). The amino acid sequence of the submitted termini, except for those originating from translational events, is summarized in an IceLogo (18), which is a frequency-based sequence logo with probability cut-off (Figure 2E). Taken together and by comparison with protease specificity logos provided by TopFIND, this can enable the identification of one or several dominant candidate protease activities in the sample. To account for the bias created by different numbers of protease-substrate associations available in TopFIND, ToFINDer also calculates enrichment statistics for each protease enabling the researcher to assess the likelihood of the protease being responsible for the cleavage. In addition to graphical representations, the statistical summary is also reported in tabular format (Table 2).

**Table 2.** Protease enrichment results from TopFINDer in the skin data set

Protease name	Protease accession	List count (total = 129)	DB count (total = 1265)	Fold enrichment	Fold coverage	Fisher exact test (*p*-value)	Adjusted Fisher exact test (*q*-value)
MMP2	P33434	68	537	1.24	0.13	1.62E-02	5.27E-02
CATE	P70269	51	544	0.92	0.09	8.03E-01	8.03E-01
THRB	P19221	1	1	9.81	1.00	1.77E-01	2.87E-01
GRAB	P04187	16	58	2.71	0.28	7.28E-04	9.47E-03
CASP3	P70677	7	28	2.45	0.25	3.67E-02	7.96E-02
CASP7	P97864	7	28	2.45	0.25	3.67E-02	7.96E-02
CATD	P18242	29	309	0.92	0.09	7.22E-01	7.82E-01
MPPB	Q9CXT8	4	4	9.81	1.00	3.65E-03	2.37E-02
CAN2	O08529	2	8	2.45	0.25	2.35E-01	3.29E-01
MMP9	P41245	5	11	4.46	0.45	1.19E-02	5.15E-02
CASP1	P29452	2	16	1.23	0.13	5.07E-01	6.00E-01
MMP8	O70138	1	2	4.90	0.50	2.53E-01	3.29E-01
MMP13	P33435	1	1	9.81	1.00	1.77E-01	2.87E-01

### PathFINDer: Protease web path-finding in TopFIND

**Figure 3.**
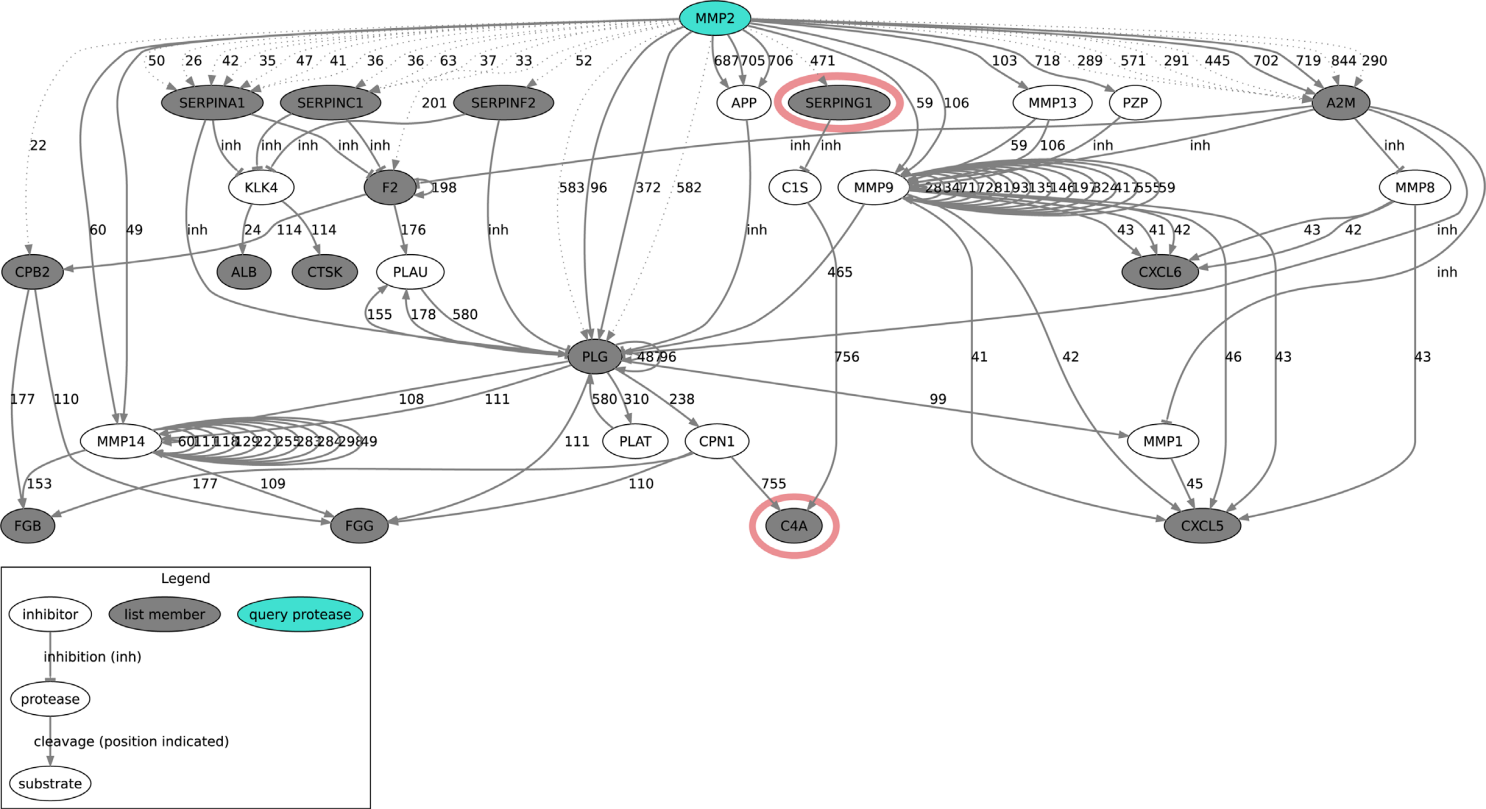
Fragment of the graphviz figure of protease web connections identified by PathFINDer. Nodes are proteins, the query protease is marked in color and the proteins from the submitted list are gray. Edges are cleavages (arrows, with numbers for the position of the cleavage) or inhibitions (T shaped arrows, labeled as ‘inh’). Edges from TopFIND are solid and edges inferred from the list are dotted. Nodes from the complement system are marked with red.

We employ path-finding in the protease web to identify known direct and indirect explanations for the observed cleavages. This powerful analysis can reveal previously hidden dependencies, facilitate differentiation of direct and indirect effects and explain counter intuitive experimental results as show previously (12). With PathFINDer it is now possible to submit a list of identified human or mouse *in vivo* substrate candidates and cleavage sites and a candidate protease to find known direct and indirect connections (paths) from the protease to the identified substrates. By creating a representation of the protease web based on cleavage and inhibition data in TopFIND and then dynamically extending this network with connections from the candidate protease to the proteins in the list, PathFINDer identifies paths from the protease to each substrate. We expanded this analysis by cross-species mapping between human and mouse orthologous proteins to compensate for the currently sparse data, in particular in mouse (12). In this way if data are absent but present for homologues in the other species a reasonable prediction can still be formulated to test. This analysis can be run separately or in combination with a TopFINDer analysis. The identified paths are visualized in a network view (Figure 3) and listed in a tabular format (Supplementary Table S3) including links to all relevant proteins, cleavages and evidences in the TopFIND web interface.

### Validation of TopFINDer

To demonstrate the utility of TopFINDer, we re-analyzed a proteomics data set of all 1255 N-termini found in inflamed and normal skin samples of wild type and matrix metalloproteinase-2 knock-out (*Mmp2-/-*) mice (7). First, the generated annotation table (Supplementary Table S1) was used to analyze the individual N-termini as to their origin (evidences) and biological impact on protein function (protein features). The Venn diagram generated by TopFINDer (Figure 2B) showed that 558 (44%) N-termini from the list correspond to UniProt annotated, canonical start sites, many of which are past the initiator methionine and map to the start of stable protein chains as defined by UniProt; 348 N-termini have experimental evidence (∼60% by TAILS), demonstrating the reproducibility of the N-terminal enrichment method TAILS; 129 N-termini are the result of cleavages, 16 of which coincide with a UniProt annotated protein start; 24 N-termini have evidence from alternative translation and 16 from alternative splicing. Thus, translation accounts for about 50% of N-termini with the other 50% likely due to proteolytic processing because cleavage data are probably far more incomplete than alternative splicing or alternative translation data. TopFINDer could identify an annotated protease for about 20% of these remaining N-termini. The histogram (Figure 2D) and table (Table 2) shows that as many as 68 cleavages could be attributed to MMP2. However, as expected when analyzing the entire list of peptides from this experiment, enrichment for MMP2 cleavages was not significant and the high count is due to the high number of known MMP2 cleavages. Interestingly, cleavages by Granzyme B and Mitochondrial-processing peptidase were overrepresented in the sample with 16 of 58 and 4 out of 4 substrate cleavages identified, respectively. Finally, the clustered substrate-protease matrix (Figure 2C) showed that there was a small overlap between MMP2 and non-metalloproteinases. Carried out manually, similar analyses that would take days or weeks to complete is returned by TopFINDer in a few minutes.

PathFINDer analysis of the proteins in the murine network so far did not yield relevant insights (data not shown) reflecting the sparse network based on murine protease–substrate association data in MEROPS and hence TopFIND (12). However, when cross-mapping to the human network, numerous connections between the candidate protease and identified substrates can be identified generating concrete and meaningful mechanistic hypotheses. For example, we observed the connection between MMP2, serpin G1 (complement inhibitor protein 1) and complement 4a, which indicated MMP2 regulatory activity on the complement system. Indeed, this was observed and validated *in vivo* by proteomic TAILS analyses that showed increased levels of cleaved serpin G1 in wild type but not *Mmp2*-/- mice in inflammation *in vivo* was also and biochemically validated *in vitro* in the original publication (3). PathFINDer thus succeeds in assembling biochemical knowledge of proteases and inhibitors and provides relevant hypotheses, which can in turn be validated experimentally.

## CONCLUSIONS

With version 3.0, TopFIND goes beyond proteolysis-generated termini and now accounts for all known biological processes that lead to variation in the start and end of a protein by assembling available evidence for termini. For any experimentally observed terminus TopFINDer thereby greatly facilitates assessment of the origin of the terminus. This is particularly powerful in combination with large lists of termini generated by current methods. The statistics of termini generating biological processes reported by TopFINDer make it possible to quickly assess biological processes in a sample. Because many termini appear to be caused by proteolytic cleavage events, TopFINDer further expands on that aspect and reports protease statistics enabling identification of active proteases in the system, allowing for the inference of biological pathways that are also active in the system. Furthermore, TopFINDer enables inference of the consequence of cleavage by reporting functional consequences of termini via domains lost and retained in a cleavage fragment remaining.

Finally, with the second termini list analysis tool PathFINDer, TopFIND 3.0 also enables the formulation of network biology inspired mechanistic hypotheses for experimental validation critical in any interpretation of *in vivo* research, where proteins can no longer be viewed as independent entities but in context as parts of a bigger interconnected complexes. Thus, TopFINDer and PathFINDer together enable powerful analysis of large proteomic data sets in addition to the existing proven web and API access methods. This database update thereby answers the need for modern, systems-biology research, which too often is slowed when new leads need to be drawn from the analysis of large protein lists. By dynamically displaying many layers of relevant data from multiple databases, TopFIND accelerates the data analysis and hypothesis generation process.

## SUPPLEMENTARY DATA

Supplementary Data are available at NAR Online.
